# Handheld optical coherence tomography–reflectance confocal microscopy probe for detection of basal cell carcinoma and delineation of margins

**DOI:** 10.1117/1.JBO.22.7.076006

**Published:** 2017-07-11

**Authors:** Nicusor Iftimia, Oriol Yélamos, Chih-Shan J. Chen, Gopi Maguluri, Miguel A. Cordova, Aditi Sahu, Jesung Park, William Fox, Christi Alessi-Fox, Milind Rajadhyaksha

**Affiliations:** a Physical Sciences, Inc., Andover, Massachusetts, United States; b Memorial Sloan Kettering Cancer Center, Dermatology Service, Department of Medicine, New York, United States; c Universitat de Barcelona, Dermatology Department, Hospital Clínic, Barcelona, Spain; d Caliber I.D., Rochester, New York, United States

**Keywords:** optical coherence tomography, reflectance confocal microscopy, basal cell carcinoma, skin cancer margins delineation

## Abstract

We present a hand-held implementation and preliminary evaluation of a combined optical coherence tomography (OCT) and reflectance confocal microscopy (RCM) probe for detecting and delineating the margins of basal cell carcinomas (BCCs) in human skin *in vivo*. A standard OCT approach (spectrometer-based) with a central wavelength of 1310 nm and 0.11 numerical aperture (NA) was combined with a standard RCM approach (830-nm wavelength and 0.9 NA) into a common path hand-held probe. Cross-sectional OCT images and enface RCM images are simultaneously displayed, allowing for three-dimensional microscopic assessment of tumor morphology in real time. Depending on the subtype and depth of the BCC tumor and surrounding skin conditions, OCT and RCM imaging are able to complement each other, the strengths of each helping overcome the limitations of the other. Four representative cases are summarized, out of the 15 investigated in a preliminary pilot study, demonstrating how OCT and RCM imaging may be synergistically combined to more accurately detect BCCs and more completely delineate margins. Our preliminary results highlight the potential benefits of combining the two technologies within a single probe to potentially guide diagnosis as well as treatment of BCCs.

## 1 Introduction

Optical coherence tomography (OCT) and reflectance confocal microscopy (RCM) are well-established optical imaging technologies, and their use, individually and independently, for the noninvasive diagnosis of nonmelanoma skin cancers (NMSCs) has been reported in several studies.^1^^–^^4^ OCT imaging is similar to ultrasound but with much higher (micron scale) resolution, with 5 to 10  μm optical sectioning and to depths of at least 1 mm.^5^ RCM imaging provides 1 to 3  μm optical sectioning and 0.5 to 1.0  μm lateral resolution, which is on par with that of conventional pathology, but the depth of imaging is limited to about 200  μm (the higher resolution is at the expense of depth).^6^

NMSCs are the most common skin malignancies in USA and in other areas of the world. Among NMSCs, basal cell carcinomas (BCCs) in particular, occur with the highest incidence rates and account for about 70% to 80% of the total NMSCs.^7^^,^^8^ Not surprisingly, several OCT and RCM imaging studies have focused on noninvasive detection of BCCs, as a possible adjunct to clinical and dermoscopic examination and pathology. Studies report sensitivities and specificities in the range 80% to 95% and 70% to 90%, respectively, for both OCT and RCM.^1^^–^^4^ Other studies have reported the ability of OCT and RCM imaging to delineate lateral margins of BCCs.^9^^–^^11^ Furthermore, studies have reported the ability of OCT to reliably detect the depth of BCCs.^12^ The ability to differentiate between superficial and deeper BCCs is clinically important because superficial BCCs can be treated with newer less invasive nonsurgical approaches. Such nonsurgical approaches include curettage-and-electrodessication, topical drug (Imiquimod) therapy, photodynamic therapy, laser ablation and/or coagulation, cryotherapy, and radiotherapy.

Since RCM provides high cellular-level resolution, it can be used to accurately detect the morphological features of BCCs and provide high diagnostic accuracy. RCM can also determine lateral margins. However, this is possible only for BCC tumors at the dermal–epidermal junction and within the papillary dermis in skin. On the other hand, OCT images deeper, into the reticular dermis, and may be used to detect deeper BCC tumors that are beyond the reach of RCM and to delineate their deep margins. Furthermore, OCT can rapidly acquire raster-stacks of cross-sectional (or, orthogonal images), whereas RCM can relatively slowly acquire en face mosaics and depth stacks. This allows for rapid OCT-guided surveillance of large volumes of tissue at relatively lower resolution followed by high-resolution RCM-guided examination of cellular-level detail at specific depths of interest. Thus, the morphologic features and margins seen in the cross-sectional (orthogonal) orientation in the OCT images can aid in the assessment of the details seen in the enface orientation in RCM images, and vice versa, producing three-dimensional (3-D) microscopic views that may prove more useful than purely two-dimensional views.

The complimentary capabilities of the two technologies may be exploited to overcome their individual limitations and provide improved detection and margin delineation of BCCs. We recently reported the combined use of OCT and RCM in a benchtop setup for imaging BCCs in skin tissue *ex vivo*.^13^ RCM was proven to be effective in detecting the presence of BCCs (mainly, features such as nests of tumor, palisading, and clefting) and delineating lateral margins in en face images. OCT was proven to differentiate between normal skin morphology and deeper BCC tumor-related features (mainly, hypoechoic dark-appearing patterns surrounded by bright-appearing stroma). Thus, our benchtop study concluded that combined RCM and OCT imaging might be beneficial for both detecting superficial and deeper nodular BCCs and delineating both lateral and deep margins.

Following the bench-top work, we proceeded to design, build, and test a handheld instrument to be used *in vivo*. In this paper, we report the integration of OCT and RCM into a handheld probe, as well as the preliminary *in vivo* evaluation of this technology for detecting the presence of superficial and nodular BCCs and evaluating their lateral margins and depth.

## 2 Methods

### 2.1 Instrumentation

**Fig. 1:**
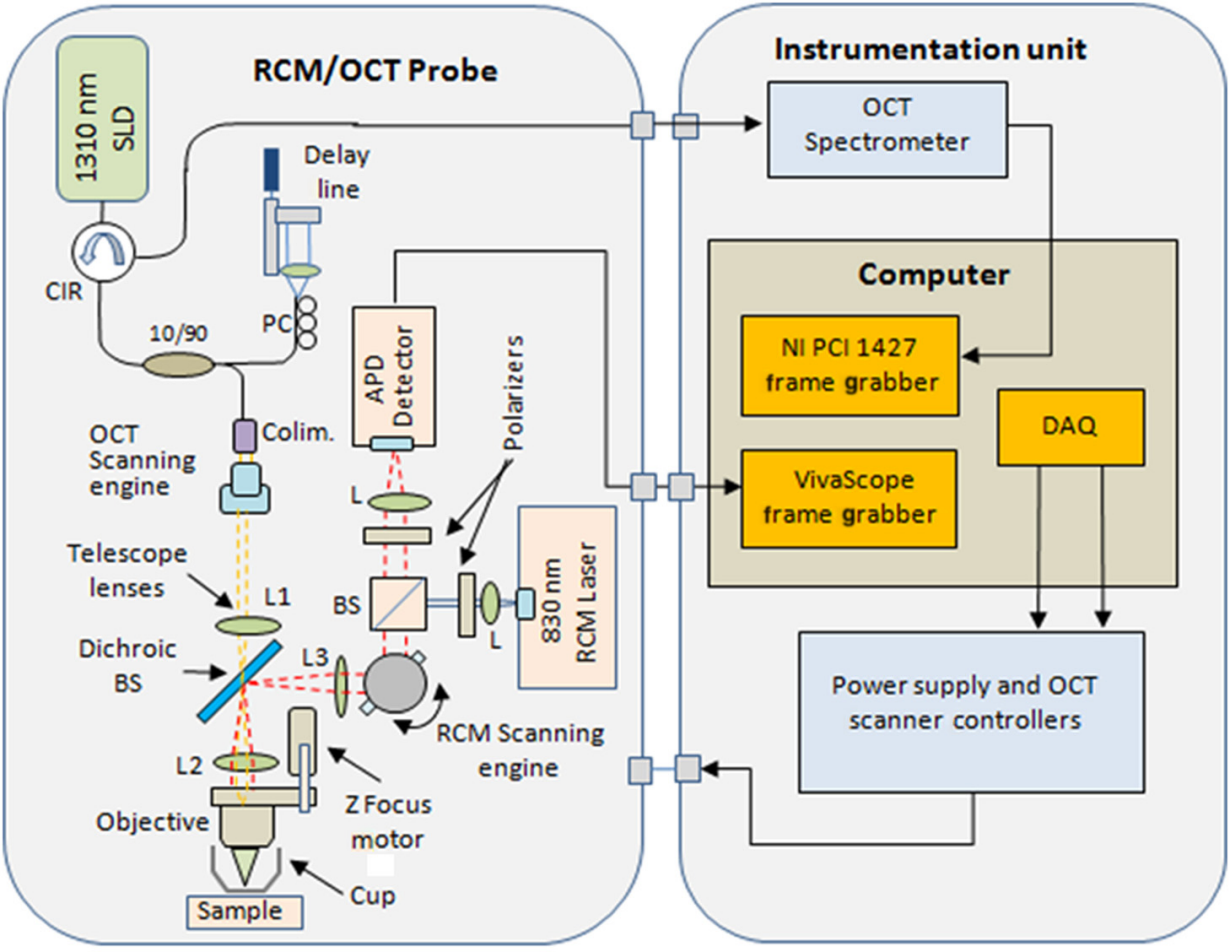
Simplified schematic of the RCM/OCT instrument.

A handheld OCT/RCM probe was developed with the goal of testing the capability of combined OCT/RCM imaging for detecting the presence of BCCs and assessing lateral and deep margins *in vivo.* A simplified schematic of the instrument is shown in Fig. 1. A T-shape telescope is used to combine OCT and RCM within the same optical path while preserving the imaging capabilities of each of the two technologies: submicron lateral resolution and optical sectioning on the order of 3  μm for RCM, and ∼1  mm imaging depth for OCT with resolution on the order of 10  μm and micron scale optical sectioning. This was possible by underutilizing the objective lens numerical aperture (NA) in the OCT mode, while fully utilizing it in the RCM mode. Specifically, the instrument uses a custom-made water immersion objective lens with an NA of 0.8. The 10-mm pupil of the lens is overfilled in the RCM mode, allowing for full utilization of the NA, and is under-filled in the OCT mode (OCT beam size is 1 mm). The theoretical lateral resolution of the RCM subsystem is about 0.53  μm (Δx=0.46λ/NA), whereas the theoretical optical sectioning is about 2.6  μm ($\Delta z=1.4n\lambda /{\mathrm{NA}}^{2}$) for a refractive index (n) of 1.33 (water immersion) and wavelength (λ) of 830 nm. The size of the OCT beam is 1 mm, and thus lateral resolution at the focus is about 7  μm (Δx=1.22  λF/D) for an effective focal length of 4.5 mm [working distance (WD) of 1.2 mm].

The focus adjustment for OCT and RCM is provided by a small stepping motor, which moves the objective lens position relative to an optically transparent cap-and-window. The cap-and-window is the only element of the probe that is in contact with the patient skin and gently flattens and stabilizes the area to be imaged. Index matching oil (n=1.55) is put on the skin surface to optically interface to the window and eliminate potential back-reflections, while ultrasound gel (n=1.34) is used inside the cup as the necessary immersion medium for the objective lens. Furthermore, the depth focusing adjustment is also used to improve OCT image quality by coadding several images acquired at several depths. This function is available in the z-stack mode, where the imaging objective focuses sequentially at different depths. Several images are coadded and processed to retrieve the maximum intensity from each depth and thus to build a sharper image with improved resolution and contrast.

The OCT subsystem consists of a fiber-optic (FO) interferometer with an optical delay line in the reference arm and a scanning engine in the sample arm. A superluminescent diode with 1310-nm central wavelength, 10-mW power, and about 100-nm 3-dB bandwidth is used as the light source. However, the power level incident to the sample is about 3.5 mW due to the inherent losses in the interferometer and optical chain. This light source provides a theoretical axial resolution of about 7.5  μm (in air). An FO circulator is used to maximize light collection from the sample arm. The OCT beam is scanned across the skin using a pair of galvanometers (Model 6200, Cambridge Technologies, Bedford, Massachusetts) to generate a raster scan or B-scan. Since each OCT frame has 512 A-lines and the OCT field of view (FOV) is 2.0 mm, about 3.9  μm/pixel lateral sampling resolution is achievable in the OCT mode. This is approximately twice smaller than the lateral resolution of the imaging objective and thus enables improved image quality.

The RCM subsystem uses an 830-nm laser light source with ∼8-mW optical power. However, due to the use of a cube splitter (see Fig. 1) and the inherent losses in the system, the power level incident to the sample is less than 4 mW. The detection unit uses an avalanche photodiode, with a pinhole that is ∼3× larger than the lateral resolution. A pinhole of diameter 3× to 5× larger than the resolution is known to reduce speckle noise, while preserving adequate optical sectioning.^14^ A set of polarizers, 90 deg rotated in respect to each other, along with a quarter wave plate, are used to minimize specular back-reflections. The fast speed RCM line scanning is performed by an 8-kHz resonant scanner (Model CRS08K, Cambridge Technologies), while the raster scan is performed by a slow galvanometer (Model 6200, Cambridge Technologies). Depending on the number of lines per scan (500 to 1000), an imaging speed of 8 to 16 Hz is possible.

The OCT and RCM beams are combined with a dichroic mirror (see Fig. 1) and sent out to the sample through the same imaging objective. However, they are not temporally colocated on the sample surface due to the use of different scanning engines, which are not synchronized. This minimizes skin exposure to less than 4 mW for a given location and eliminates any potential for skin damage.

Both the OCT and the RCM reflected signals are detected, digitized, and then processed by a data processing unit, consisting of a graphical processing unit (GPU, model GT 740 which uses 384 CUDA cores) for the OCT signal and a custom-made frame grabber (Model VivaGrab, Caliber I.D.) for the RCM signal. The GPU is used to expedite the OCT data processing [fast fourier transform, dispersion, and interpolation], allowing for 60  frames/s real-time display. The RCM imaging speed is mainly dictated by the speed of the resonant scanner and the processing capability of the custom-made frame grabber.

**Fig. 2:**
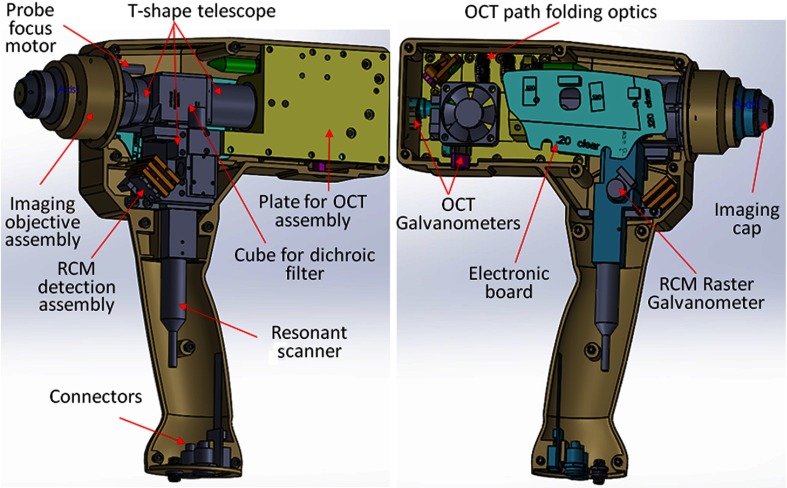
Solid Work (CAD) design of the handheld OCT/RCM probe.

The feasibility of implementing the OCT-RCM technology within a handheld probe was first evaluated by performing computer-aided design (Solid Works). As observed from Fig. 2, a small footprint, just slightly larger than that of a typical hair dryer, was possible using custom-made optics and optomechanical parts. The dichroic mirror combines the OCT and RCM optical paths was placed inside a cube, in which the OCT and the RCM assemblies were attached at the input ports and the imaging objective at the output port. A custom-designed electronic board was used to control the RCM and OCT scanners, as well as to amplify and digitize the RCM signal.

### 2.2 Instrument Fabrication, Testing, and Optimization

**Fig. 3:**
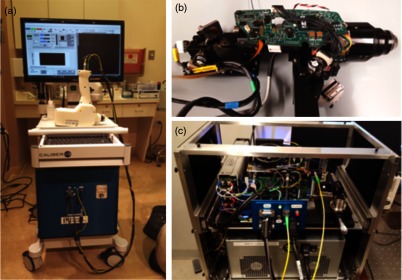
Photographs of the RCM/OCT imaging instrument: (a) general view of the instrumentation unit and hand-held probe; (b) probe detail; and (c) instrumentation unit detail.

The OCT/RCM instrument was fabricated by our team at Physical Sciences, Inc. in collaboration with Caliber I.D. Most of the optomechanical and electrical parts for the probe and instrumentation unit were custom-fabricated. The instrumentation unit was placed onto a mobile cart, while the probe was enclosed into a 3-D-printed shell. Photographs of the instrument and hand-held probe are shown in Fig. 3. The instrumentation unit and the probe were fully tested and optimized before performing measurements on patients. Coregistration of the RCM and OCT images, imaging depth, sensitivity decay within the imaging range, FOV, and imaging resolution were optimized.

OCT-RCM image coregistration was first tested using an USAF 1951 positive resolution target, with the objective that the center of the RCM image must correspond to the center of the enface OCT image. For this purpose, enface OCT and RCM images were taken and the offsets were applied to the OCT beam to coalign it with the RCM beam. The lateral resolution in both modes was determined as well using the USAF 1951 target. A lateral resolution of about 5  μm was measured in the OCT mode, whereas the RCM resolution was better than 1  μm.

The axial resolution of OCT was evaluated by measuring the full width at the half maximum of the coherence peak. The theoretical axial resolution ($\delta z=0.44\lambda_{0}^{2}/\Delta \lambda$, where $\lambda_{0}$ is the central wavelength of the OCT light source and Δλ is its 3-dB bandwidth), of the instrument is about 7.5  μm (in air). However, a value of 8  μm in air, which corresponds to ∼6  μm in tissue, has been measured. The difference is thought to be attributed to slight clipping of the spectrum on the spectrometer camera, and thus the full bandwidth of the source has not been used.

The maximum FOV is 2.0 mm in the OCT mode and 0.8 mm in the RCM mode. The OCT FOV is mainly limited by the imaging objective. A larger FOV will require the use of an objective with a larger entrance pupil and a higher WD. Unfortunately, a longer WD would require an objective lens with a lower NA, which would then reduce the optical sectioning and resolution in the RCM mode.

The measured imaging speed was 8  frames/s (1024×1024  pixels) in the RCM mode and 60  frames/s (1024×1024  pixels) in the OCT mode. However, the OCT display rate was decimated to 30  frames/s to preserve GPU resources for processing the OCT data in real time.

### 2.3 Imaging Protocol

A preliminary evaluation of the RCM/OCT device was performed at Memorial Sloan Kettering Cancer Center (MSKCC) under an Institutional Review Board-approved protocol (IRB #: 99-099 A24). Patients with skin lesions either diagnosed as BCC after biopsy or clinically/dermoscopically suspicious for BCC were identified by their physicians and offered an opportunity to participate in this study.

Following informed and written consent, the suspicious area was clinically (visually) assessed using surface epiluminescent microscopy (also known as dermoscopy), as per routine practice. A sterile cap-and-window device was used on patients, as a mechanical interface between the probe’s objective lens and the skin. This window is placed in contact with skin surface, gently flattening and stabilizing the area to be imaged. To facilitate navigation of the OCT/RCM probe during imaging, an adhesive paper ring with an internal diameter of 10 mm was placed on the skin with the identified lesion located within the center of the ring. Then, a drop of index-matching mineral oil was applied. The application of oil provides refractive index matching between the window and skin and diminishes specular back-reflection from the skin surface. The paper ring was used as a reference to ensure that the probe remains consistently positioned on the site of interest.

Imaging was performed in two distinct modes: (a) simultaneous stacks of OCT and RCM images with the objective lens focus starting from the tissue surface (stratum corneum) to a depth of 250  μm, with 5-μm spacing and (b) video RCM and OCT imaging (raster mode) with the objective lens focus kept at a single depth (usually, 150  μm below the tissue surface). While imaging, the first goal was to identify typical features of tumor nests with RCM, such as palisading and clefting, and then to evaluate the depth spreading with OCT. After localizing tumor nests, both z-stack and rasters were collected to enable the acquisition of multiple images that were postprocessed to further evaluate tumor lateral and deep margins.

### 2.4 Preparation of Histopathology and Comparison With RCM/OCT Images

After OCT/RCM imaging, the lesions were surgically removed, either using shave excision or margin-controlled Mohs surgery, as per routine practice. The histopathologic specimens obtained after excision were compared to the OCT/RCM images. We first calculated the deep margins of BCC tumors from the OCT images using LabView-Based software that allows for placing two markers, one at the tissue surface and one at the desired depth. Then, we measured the tumor depth from the histology slides. RCM enface images and cross-sectional/enface OCT images were analyzed to identify features of BCCs and evaluate the lateral and deep margins. The RCM typical features of BCCs were cord-like/nodular structures with peripheral palisading and/or clefting admixed with a fibrotic stroma, whereas OCT typical features were dark hypoechoic areas, in some cases surrounded by a stronger signal, aura-like, caused by the high scattering of the stromal tissue around the tumor nodules.

## 3 Results

OCT/RCM measurements were performed on 15 lesions occurring in 10 patients. BCC-suspicious features were identified with dermoscopy in all 15 lesions. However, BCC features were identified in only 12 cases with the OCT/RCM probe. In the remaining three cases, RCM and OCT indicated the absence of BCCs, and histology confirmed these findings. Out of the 12 BCC positive cases, both RCM and OCT clearly identified BCCs in nine cases, whereas in two cases, RCM identified BCCs, which was suspected but not clearly evident with OCT, and in one case, RCM was suspicious for a BCC that was unequivocally identified in the OCT mode. Histology confirmed the presence of BCCs in all 12 cases. Herein, we summarize four representative cases for which histology was used to correlate imaging findings.

**Fig. 4:**
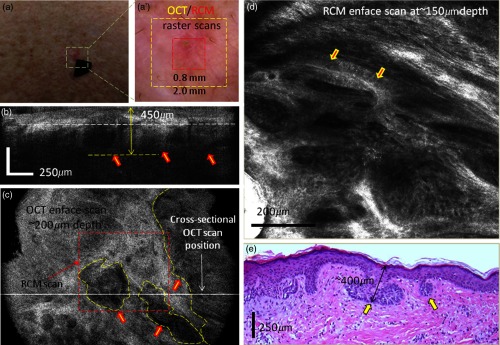
(a) Clinical image showing an erythematous macule on the back. (a′) Dermoscopy image showing shiny white lines and serpentine vessels. (b) Cross-sectional and (c) enface OCT images showing multiple hypoechoic areas (see arrows), suggestive of BCC. (d) Reflectance confocal microscopy showing cord-like structures with peripheral palisading (arrows) admixed with a fibrotic stroma, suggestive of BCC. (e) Histology image of the lesion (confirming the diagnosis of superficial BCC), showing multiple small tumor nests originating from the epidermis (hematoxylin and eosin, 4× magnification).

*Case 1: Both RCM and OCT confirmed presence of BCC and OCT further allowed for accurate margin delineation*. This case is of an erythematous macule on the back of a male patient [Fig. 4(a)]. On dermoscopy, this was a typical case of a BCC showing shiny white lines and serpentine vessels [see Fig. 4(a′)]. Both cross-sectional OCT and enface RCM imaging simultaneously revealed the features of the BCC. RCM showed cord-like structures with peripheral palisading admixed with a fibrotic stroma [see arrows in a representative image in Fig. 4(d)], which is a common feature for superficial BCC.^1^^,^^2^ The cross-sectional OCT images showed hypoechoic areas in the upper dermis along the dermal-epidermal junction [see arrows in the selected frame in Fig. 4(b) out of the 512 collected frames], which are characteristic of superficial and early nodular BCCs. The enlarged and densely packaged nuclei (i.e., increased nuclear volume relative to cellular cytoplasm) in the tumor area scatter light weakly, giving rise to a hypoechoic area relative to the surrounding strongly scattering bright-appearing stroma in the OCT image. These tumor islands are well-demarcated with our probe. They can be detected as deep as 700  μm from the skin surface. The maximum depth in this case was evaluated to be ∼450  μm by OCT and was confirmed by histology, when the shrinkage effect during histology processing was taken into account [see Fig. 4(e)].^15^ The demarcation of the lateral margins for the individual nodules was provided by the enface OCT images, which were obtained by performing 3-D rendering of the 512 collected OCT frames (2.0  mm×2.0  mm raster scan). A typical enface image selected from the 3-D data cube is shown in Fig. 4(c). This frame corresponds to a 200-μm depth from the skin surface [corresponding to the white dotted line shown in Fig. 4(b)] and was selected to show the enface OCT appearance of some of the BCC nests (see manually segmented areas in the yellow-colored dashed outlines) surrounded by bright-appearing collagen bundles. The hypoechoic areas demarcate very well the lateral margins of the individual tumors.

A histology slide, shown in Fig. 4(e), confirms the diagnosis of a superficial BCC detected by both OCT and RCM, showing multiple small tumor nests “budding off” from the epidermis into the dermis.

**Fig. 5:**
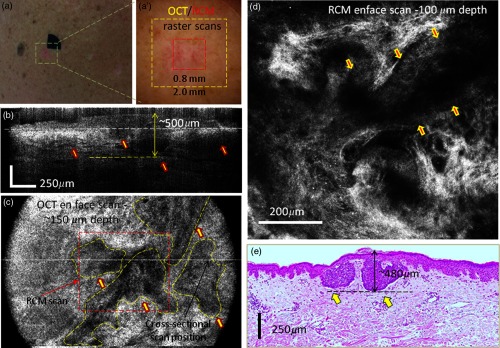
(a) Clinical image showing an erythematous macule on the right shoulder. (a′) Dermoscopy image showing shiny white lines and serpentine vessels, suggestive of superficial BCC. (b) Cross-sectional and (c) enface OCT images showing multiple hypoechoic areas, suggestive of BCC. (d) Reflectance confocal microscopy showing cord-like structures with palisading (arrows) surrounded by reticulated collagen and inflammatory cells. (e) Histology showing multiple tumor nests with different sizes originating from the epidermis (arrows) (hematoxylin and eosin, 4× magnification).

*Case 2: OCT identified areas suspicious for BCC, which were confirmed with RCM.* This is the case of an erythematous macule on the right shoulder of a female patient, showing similar clinical and dermoscopic features of a superficial BCC as in the first case [see Figs. 5(a) and 5(a′)]. The cross-sectional and enface OCT images showed in this case hypoechoic areas below the epidermis with slightly lower contrast than in the previous case [well defined in the center but hardly seen at the left and right sides of the image in Figs. 5(b) and 5(c)], making these areas suspicious for BCC nests but not clearly enough to render an unequivocal diagnosis. On the other hand, RCM shows cord-like structures with palisading (arrows) surrounded by reticulated collagen and inflammatory cells, which are clear indicators for the presence of BCC [Fig. 5(d)]. Histologically, the lesion showed multiple tumor nests with different sizes originating from the epidermis, consistent with a superficial BCC [see arrows in Fig. 5(e)]. This case clearly shows the limited specificity of OCT and the need for RCM as a confirmatory tool.

**Fig. 6:**
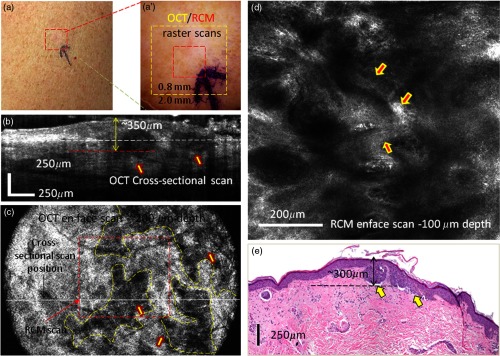
(a) Clinical image showing a subtle whitish macule on the left arm. (a′) Dermoscopy image showing shiny white lines and few serpentine vessels. (b) Cross-sectional and (c) enface OCT images show multiple hypoechoic areas suggestive of BCC. (d) Reflectance confocal microscopy image showing cord-like structures without clear palisading (arrows). (e) Histology image of the lesion revealing multiple small tumor nests originating from the epidermis confirming the diagnosis of superficial BCC (hematoxylin and eosin, 4× magnification).

*Case 3: RCM identified an area suspicious for BCC, which OCT confirmed.* This is the case of a female patient with a lesion on the left arm. Clinically, the lesion showed a subtle whitish macule [see Fig. 6(a)]. Dermoscopically, this lesion showed discrete shiny white lines and few serpentine vessels, which were suggestive for superficial BCC [see Fig. 6(a′)].

RCM showed cord-like structures without clear palisading, which were suggestive but not diagnostic for BCC [Fig. 6(d)]. Cross-sectional OCT showed multiple hypoechoic areas, suggesting the presence of BCC. A representative frame is shown in Fig. 6(b). As observed, two hypoechoic areas are easily detectable. The enface OCT image shown in Fig. 6(c), taken at a depth of ∼200  μm from the tissue surface [corresponding to the dotted white line in Fig. 6(b)], shows multiple of such hypoechoic areas, suggesting BCC nests scattering over almost the entire imaged area. A biopsy of the lesion [Fig. 6(e)] revealed multiple small tumor nests situating along the dermal-epidermal junction, confirming the presence of a superficial BCC. This case shows the potential benefit of the combined imaging modality, where the use of OCT helped to confirm the diagnosis that was suspected by RCM.

**Fig. 7:**
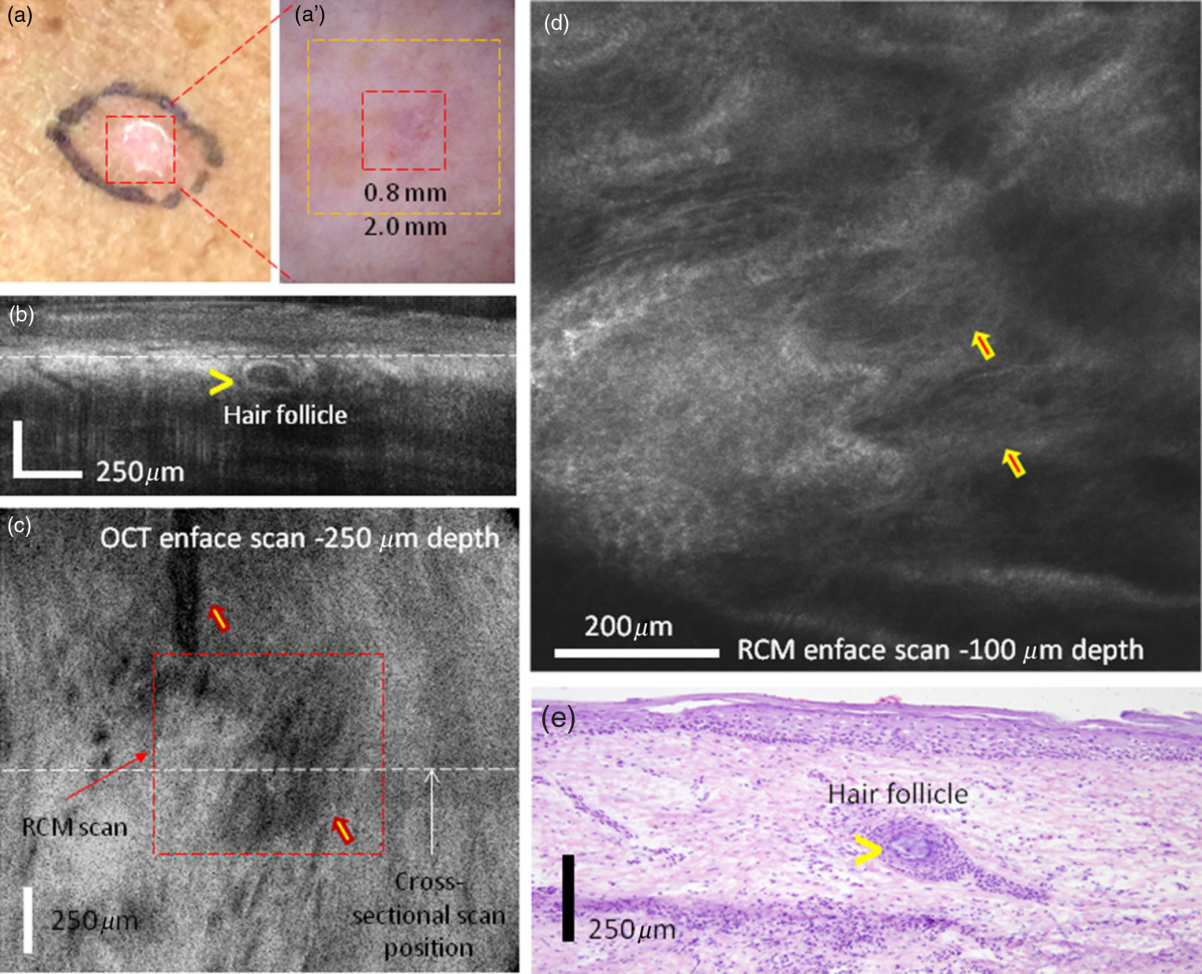
(a) Clinical image showing a subtle whitish macule on a female’s leg. (a′) Dermoscopy image showing subtle white lines, rainbow pattern, and a few vessels. (b) Cross-sectional and (c) enface OCT images showed hypoechoic areas corresponding to branched blood vessels, and a hypoechoic nodular area surrounded by a hyper-reflective halo corresponding to a hair follicle. (d) Reflectance confocal microscopy image showing bundled collagen (arrows) but no cordlike structures suggestive of BCC. (e) Histology image of the lesion revealing a hair follicle (see arrowhead highlighting the hair) and bundled collagen corresponding to fibrosis secondary to trauma (hematoxylin and eosin, 4× magnification).

*Case 4: RCM and OCT both excluded the presence of a clinically suspicious BCC.* This is the case of a female patient with a suspicious lesion on the leg. Clinically [Fig. 7(a)], she presented with a subtle pink macule. Dermoscopically, this lesion showed a few shiny white lines, discrete rainbow pattern and very few vessels, which are suggestive of a superficial BCC but can also indicate a scar [see Fig. 7(a′)].

RCM showed a normal honeycombed pattern in the epidermis [Fig. 7(d)] and bundled collagen fibers, suggesting tissue fibrosis caused by previous trauma. Cross-sectional OCT imaging (512 frames) showed normal tissue morphology and a darker structure starting from deep dermis and protruding into the epidermis, which is the typical OCT appearance of a hair follicle. A representative frame is shown in Fig. 7(b). As observed, the darker structure is surrounded by a white rim, clearly indicating the morphology of a hair follicle [yellow arrowhead in Fig. 7(b)]. The enface OCT image from Fig. 7(c), taken at a depth of ∼250  μm from the skin surface [corresponding to the dotted white line in Fig. 7(b)], shows an elongated dark structure, which might be caused by the angled positioning of the hair follicle [see yellow arrow near the center of Fig. 7(c)], as well as a very dark elongated and tortuous appearing structure, which is a clear indication of the presence of a capillary vessel [see upper yellow arrow in Fig. 7(c)]. The biopsy did not reveal the presence of BCC, confirming the OCT and RCM findings [see histology slide in Fig. 7(e), 4× magnification]. This case shows the potential benefit of the combined imaging modality, which helped exclude the presence of BCC and confirmed the presence of a scar.

## 4 Discussion and Conclusion

Our pilot study has demonstrated the feasibility of using combined OCT and RCM within a single optical layout to determine the presence of superficial and nodular BCCs *in vivo*, as well as the demarcation of lateral and deep margins. OCT has proven to provide cross-sectional images with structural-level resolution, to depths of about 1.0 mm, and to differentiate between the normal skin morphology and BCC tumor-related features: formation of round-shaped hypoechoic areas, often surrounded by a stronger signal, aura-like, caused by the high scattering of the stromal tissue around the tumor nodules. 3-D rendering of the OCT data allowed for building enface images at various depths that can be further processed (segmented) to demarcate the lateral and the deep margins of BCCs. RCM has proven to be effective in providing enface images with cellular-level resolution in superficial skin, to depths of about 200  μm, thus allowing for accurate detection. However, due to its limited penetration depth, it could not be used to determine deep margins in tumors extending beyond 200  μm.

Our results suggest that OCT/RCM images correlate well with histology and thus can potentially allow adequate margin delineation of BCC, especially the deeper margins. The combined use of RCT and OCT seems to have multiple advantages as opposed to using RCM alone or OCT alone: OCT images the volume of the skin lesion very quickly with a stack of orthogonally oriented images, each of FOV 2 mm across by 1.0-mm deep. OCT imaging detects dark hypoechoic areas, which indicate the potential presence of BCC. However, dark hypoechoic areas under OCT can also correspond to other confounding structures such as optically empty spaces within the collagen network in dermis, hair follicles, sebaceous glands, or clusters of inflammatory cells. Therefore, adding RCM imaging to examine such areas offers the advantage of evaluating the area at the cellular level and thus excluding or confirming tumor presence by identifying BCC features such as tumor nests, palisading, and clefting. Furthermore, another key advantage of our combined probe is that it can provide precise delineation of the depth of the tumor.

We observed that, depending on the surrounding skin conditions, OCT and RCM imaging are able to simultaneously complement each other, the strengths of each helping overcome the limitations of the other. In relatively pristine skin, RCM imaging, due to its cellular-level resolution, can detect the presence of BCCs in enface optical sections, while, in relatively scarred or degraded skin (due to, for example, a previous biopsy or surgery or due to photodamage), RCM imaging cannot. However, OCT can, due to its imaging being deeper and in cross-sectional (or orthogonal) optical sections. Both RCM and OCT imaging can determine the lateral margins of superficial and nodular BCCs in enface optical sections, while OCT can further detect deep margins in cross-sectional sections. Thus, with the combined technical capabilities of the probe, the RCM/OCT imaging approach can, in the future, potentially provide clinically useful imaging to guide diagnose and also to measure margins of BCC tumors to guide therapy in diverse skin conditions.

Beyond this pilot study, the next phase of technical work will be to further improve the probe and the data processing software. In particular, we will develop an automated segmentation algorithm that might be used to more objectively assess the depth of BCC tumors. We are currently improving both the real-time data collection and the postprocessing software to enable improved image quality and automated 3-D rendering of the collected data. The new software will provide better contrast in the OCT reflectance mode (with reduced speckle noise) and increased imaging depth (over 1.2 mm) by processing a series of images collected with the objective lens focusing at different depths. The next phase of clinical work will be a larger study to perform reading and interpretation of the diagnostic features, with rigorous statistical correlation to pathology, and to determine the accuracy for diagnosis and determination of depth. With continued development, testing, and refinement, we expect our integrated multimodal RCM and OCT imaging approach to overcome the individual limitations of each modality by providing 3-D microscopic views in orthogonally oriented and enface-oriented planes with a range of resolutions and fields of view, which should further advance optical imaging to noninvasively guide both diagnosis as well as therapy.
